# Supplementary material for: BADLOCK: A Rapid, Portable, Inexpensive Diagnostic for Bacterial Pathogen and Resistance Detection in Resource-Limited Settings
Source: medRxiv. 2025 Aug 13:2025.08.11.25332217. Preprint. [Version 1] doi: 10.1101/2025.08.11.25332217 (PMC12363746; doi:10.1101/2025.08.11.25332217)
Supplement: Supplementary file 1 [file media-1.pdf]

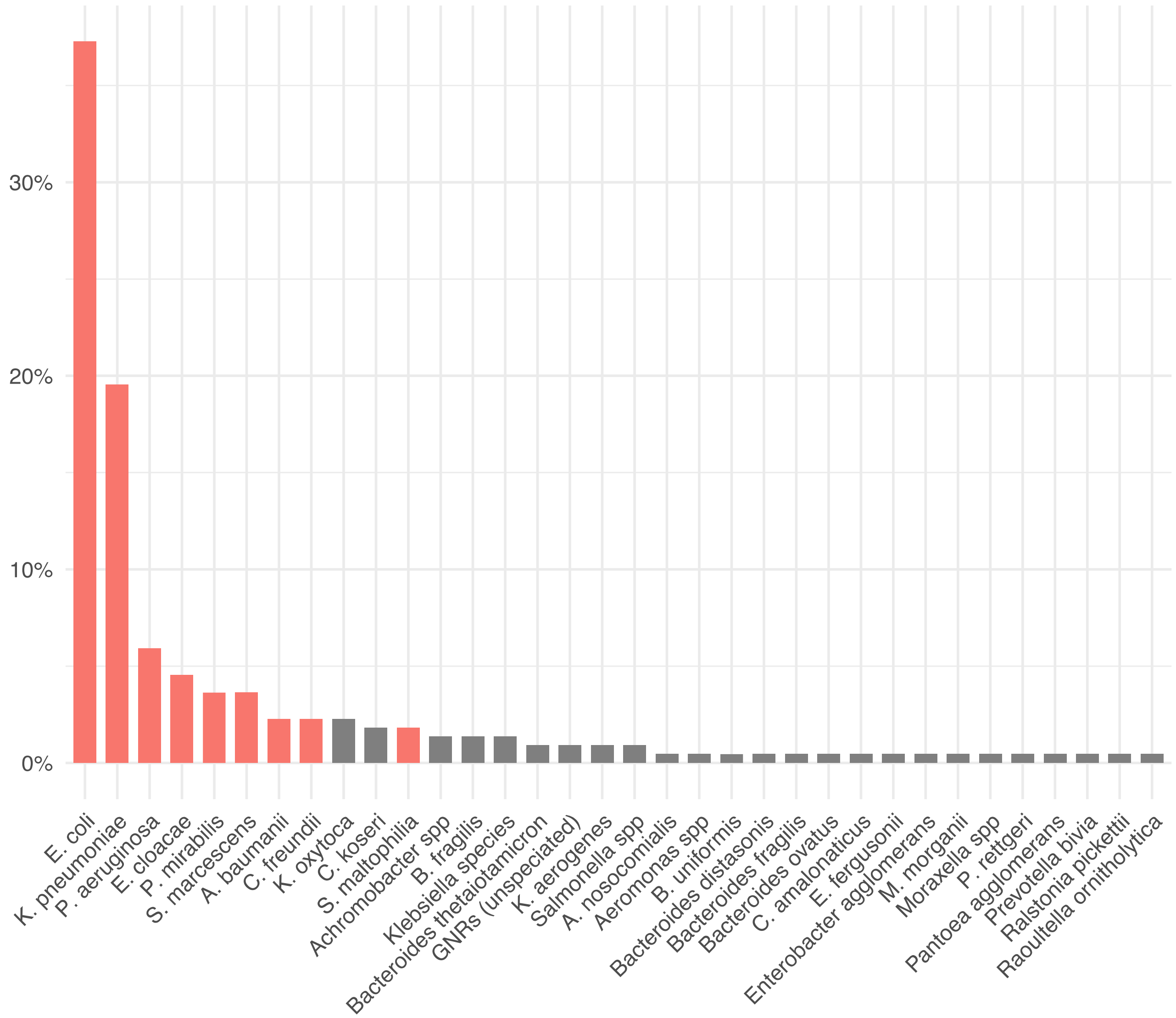

Figure S1. Proportional representation of gram-negative rod species from our entire clinical cohort. Species included in BADLOCK are shown in red.

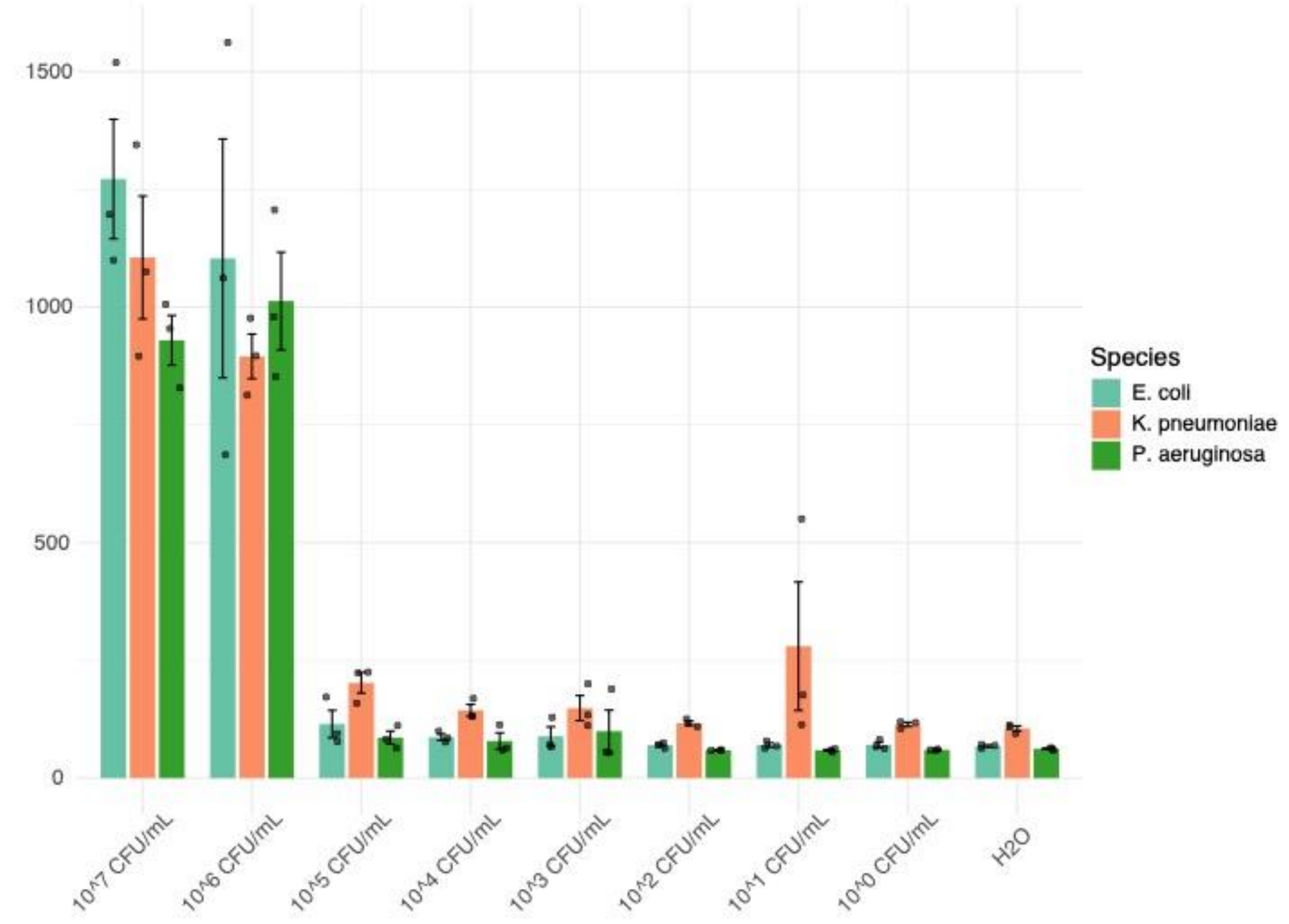

Figure S2. Limit of detection (LOD) analysis of the BADLOCK assay using LB-cultured isolates. RFU values are shown for three representative bacterial species across a serial dilution range measured in CFU/mL. The BADLOCK assay reproducibly detects targets at concentrations of ~10^6 CFU/mL (corresponding to 10^3 CFU/uL), establishing this as the approximate lower limit of detection under these experimental conditions.

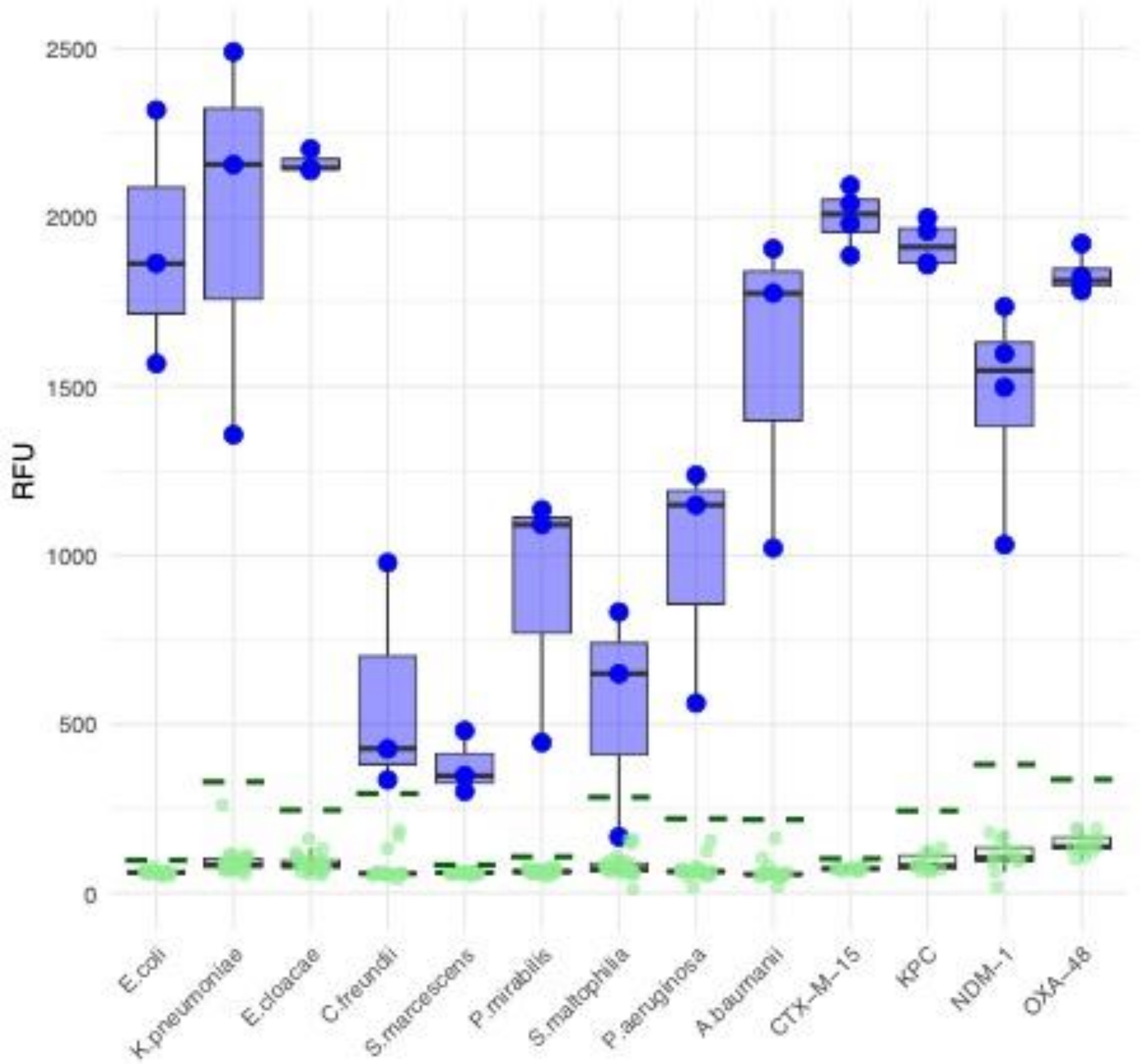

Figure S3. Threshold determination for species and antimicrobial resistance gene targets in the BADLOCK assay. Fluorescence values from technical replicates are shown for species targets using clinical samples, and for antimicrobial resistance (AMR) gene targets using banked clinical strains. The horizontal dashed line indicates the threshold for calling a positive result, defined as the mean plus six times the standard deviation of fluorescence values from 24 off-target samples for species reactions and 12 off-target samples for AMR gene reactions.

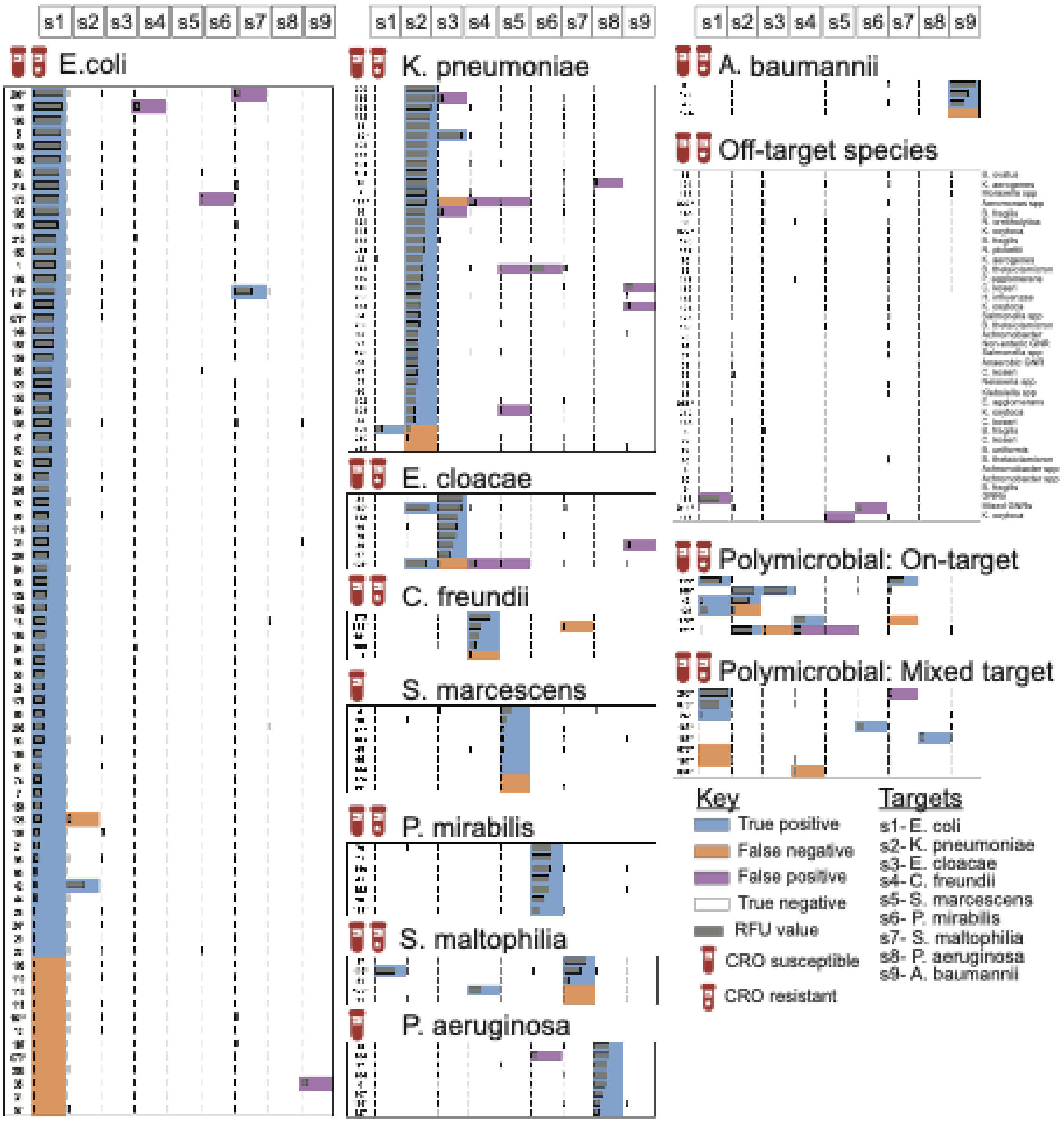

Figure S4. Consolidated visualization of BADLOCK species assay runs across all tested blood culture samples collected for this study. Each row represents an individual sample, and each column corresponds to a specific BADLOCK target with the assay layout shown in the boxes above and enumerated in the accompanying key. Samples are grouped by the species for which they were known positives as identified by the clinical microbiology laboratory's standard workflow. Each cell in the grid reflects a single target reaction for a given sample, color-coded by accuracy. Overlaid grey bars represent the corresponding RFU values. Symbols adjacent to species names indicate the cohort origin of each sample and are harmonized with the legend in main-text Figure 3. Only the first run for each reaction is shown here. Polymicrobial samples appear multiple times - once for each on-panel species present, and again in the "polymicrobial" section.

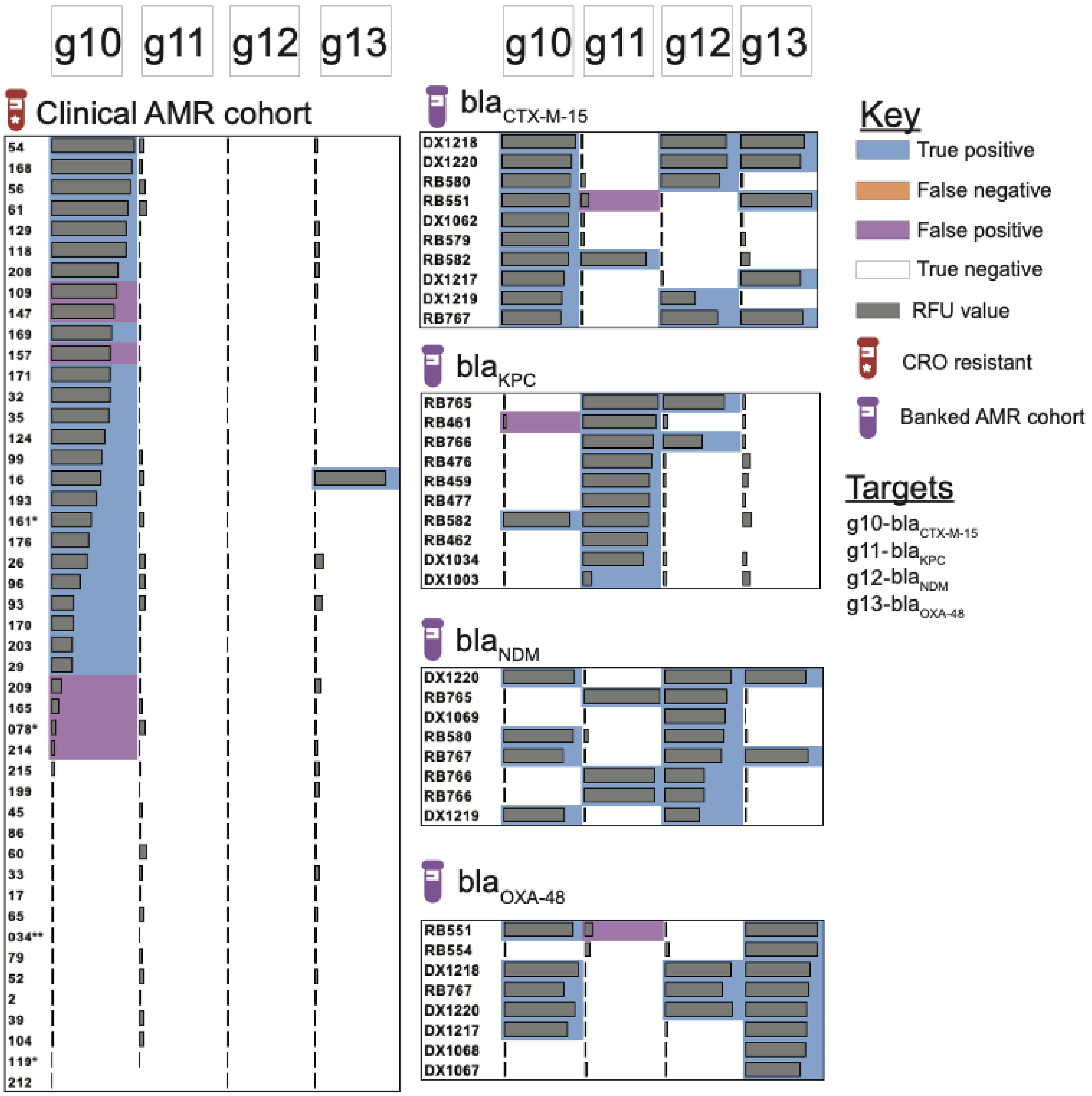

Figure S5. Consolidated visualization of BADLOCK AMR gene assay runs across all tested samples. Each row represents an individual sample, and each column corresponds to a specific BADLOCK AMR gene target, with the assay layout shown in the boxes above and enumerated in the accompanying key. Samples are grouped by the resistance gene known to be present by either PCR testing (clinical resistant cohort) or whole genome sequencing (banked cohort) (Table S3). Each cell in the grid reflects a single target reaction for a given sample, color-coded by accuracy, with overlaid grey bars representing the corresponding RFU values. Symbols adjacent to gene names indicate the cohort origin of each sample and are harmonized with the legend in main-text Figure 3. Samples containing multiple AMR gene targets appear in each group for which a corresponding target was known to be present.

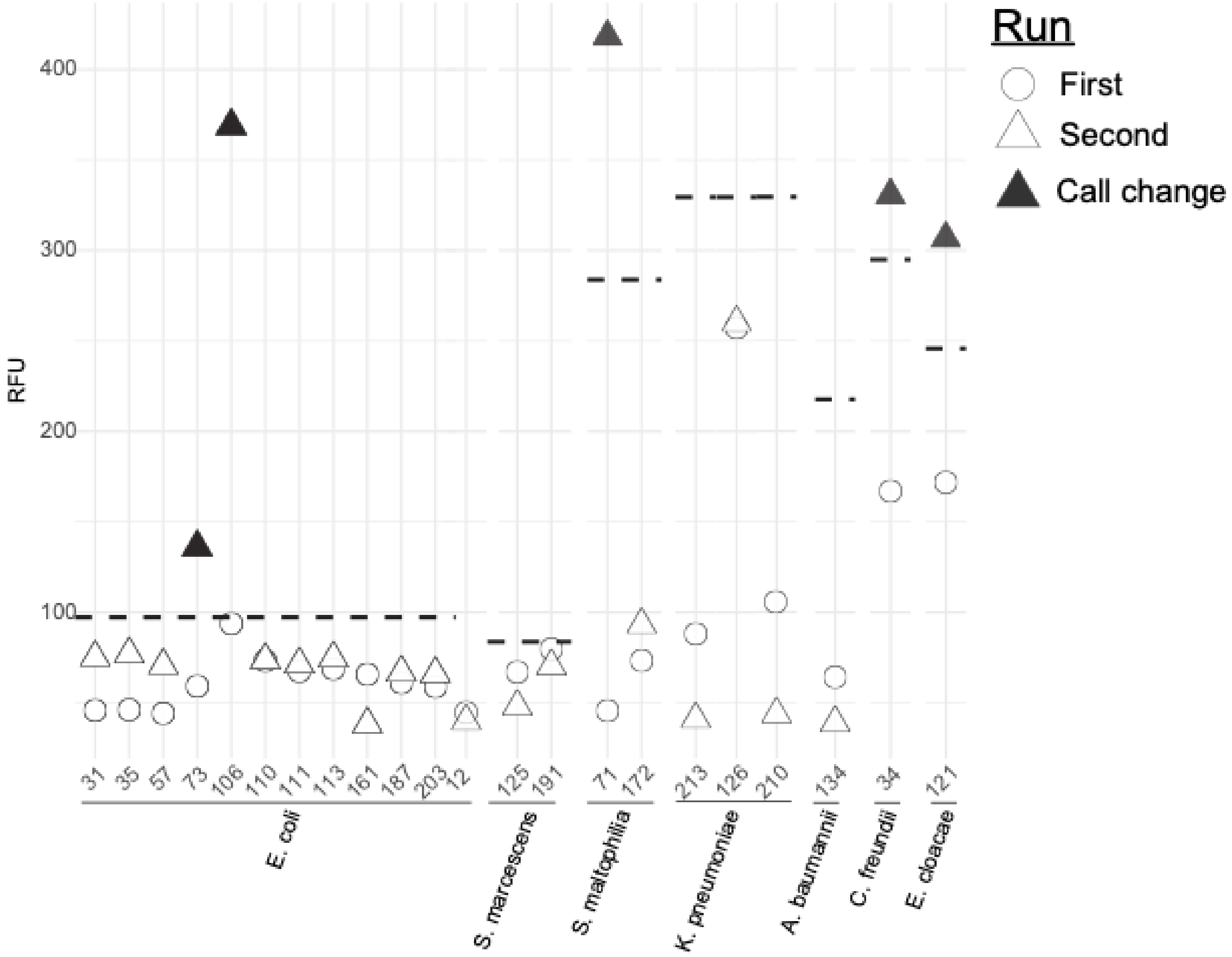

Figure S6. Repeat testing of falsely negative samples in the BADLOCK assay. Each point represents a clinical sample that was initially negative by BADLOCK but positive for the indicated species by the reference method. For simplicity, only the discordant targets are shown. Samples are displayed along the x-axis, grouped by species, with shape indicating whether the data correspond to the original or repeat run. The dashed horizontal line marks the species-specific RFU positivity threshold. A change in result classification occurs when a point crosses this threshold between runs, marked by a shaded triangle. 5 samples retested as concordant, while 17 remained discordant. Ten of these discordant negatives were in *E. coli* while 7 were non-*E. coli* species, and 4 of these were from polymicrobial samples (IDs 172, 126, 34, and 121). One *E. coli* sample that retested as concordant was 106, which was confirmed to have the *chuA* gene target by PCR, while the other lacked the target.

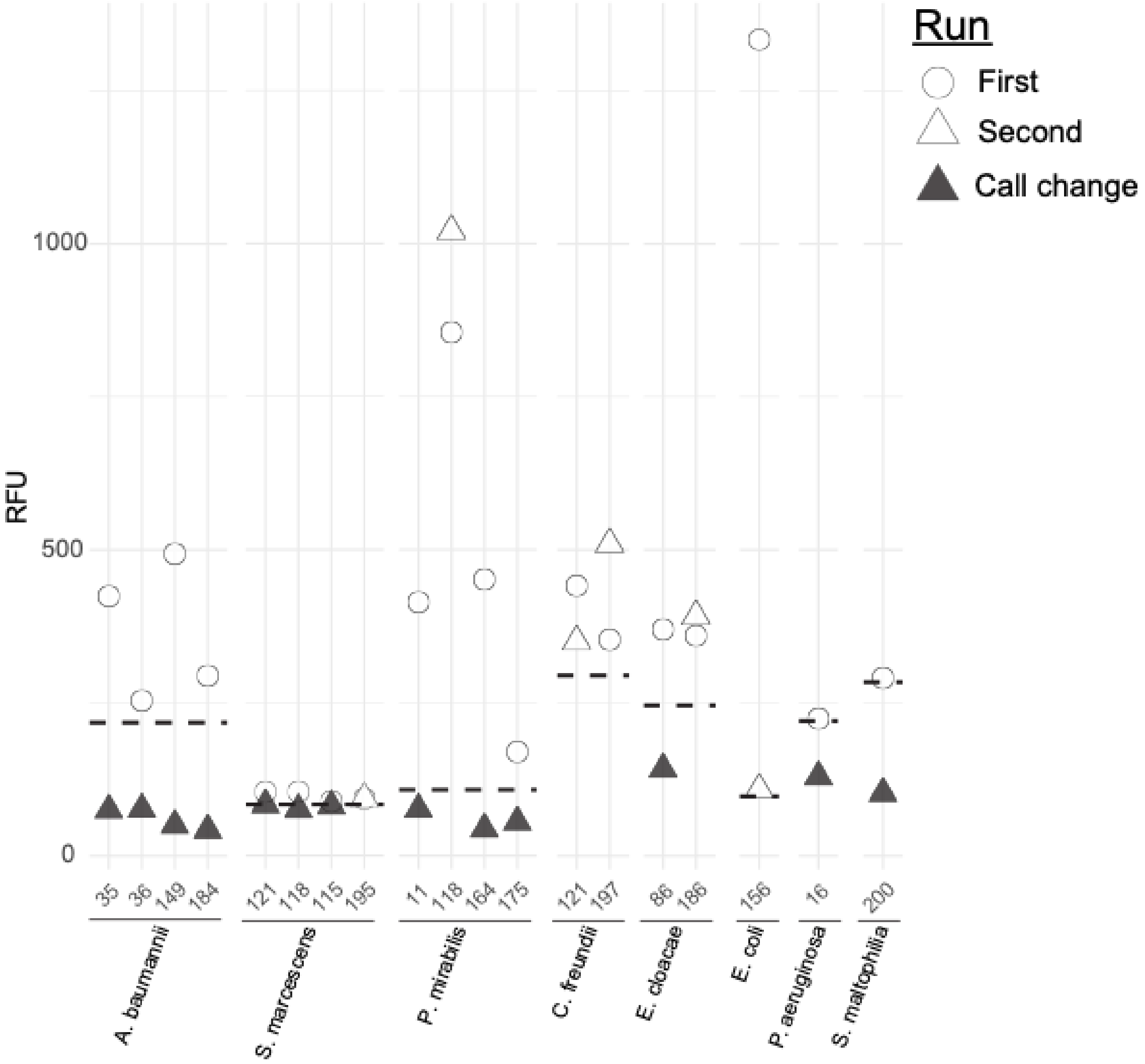

Figure S7. Repeat testing of discordantly positive samples in the BADLOCK assay. Each point represents a clinical sample that was initially positive by BADLOCK for the indicated species, which was not found by the reference method. For simplicity, only the discordant targets are shown. Samples are displayed on the x-axis and grouped by species, with shape distinguishing original versus repeat reactions. The dashed horizontal line indicates the species-specific RFU positivity threshold. A change in result classification occurs when a point crosses this threshold between reactions, marked by a shaded triangle. Upon retesting, 13 samples were corrected, whereas 7 remained discordant. Of these, two samples (IDs 195 and 156) yielded RFU values within 11% of the threshold in both reactions, whereas the remaining four consistently showed strong positive signals above threshold.

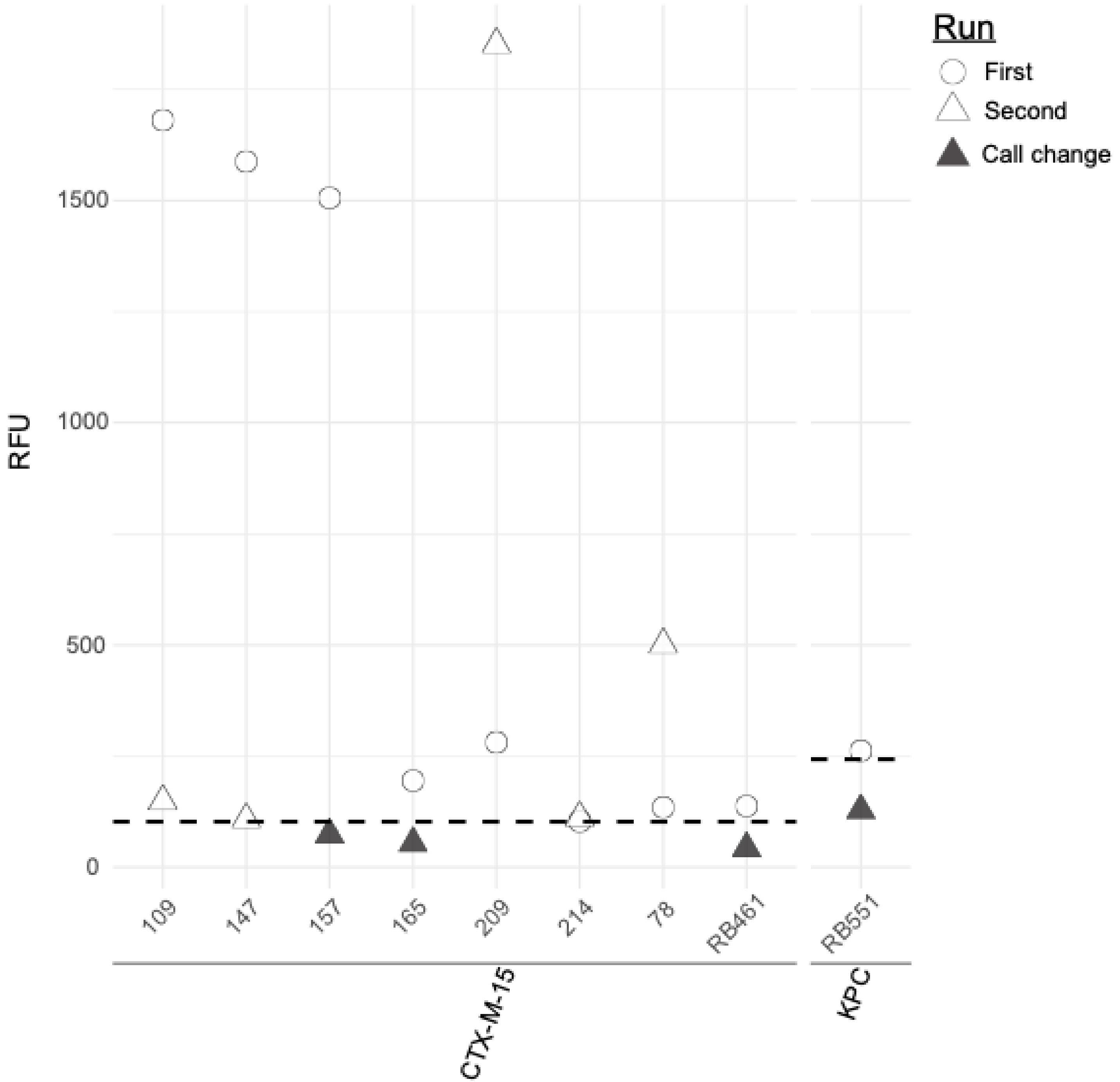

Figure S8. Repeat testing of discordantly positive samples in the BADLOCK AMR assay. Samples are displayed on the x-axis and grouped by AMR target, with shape distinguishing original versus repeat reactions. The dashed horizontal line indicates the target-specific RFU positivity threshold. A change in result classification occurs when a point crosses this threshold between reactions, marked by a shaded triangle.

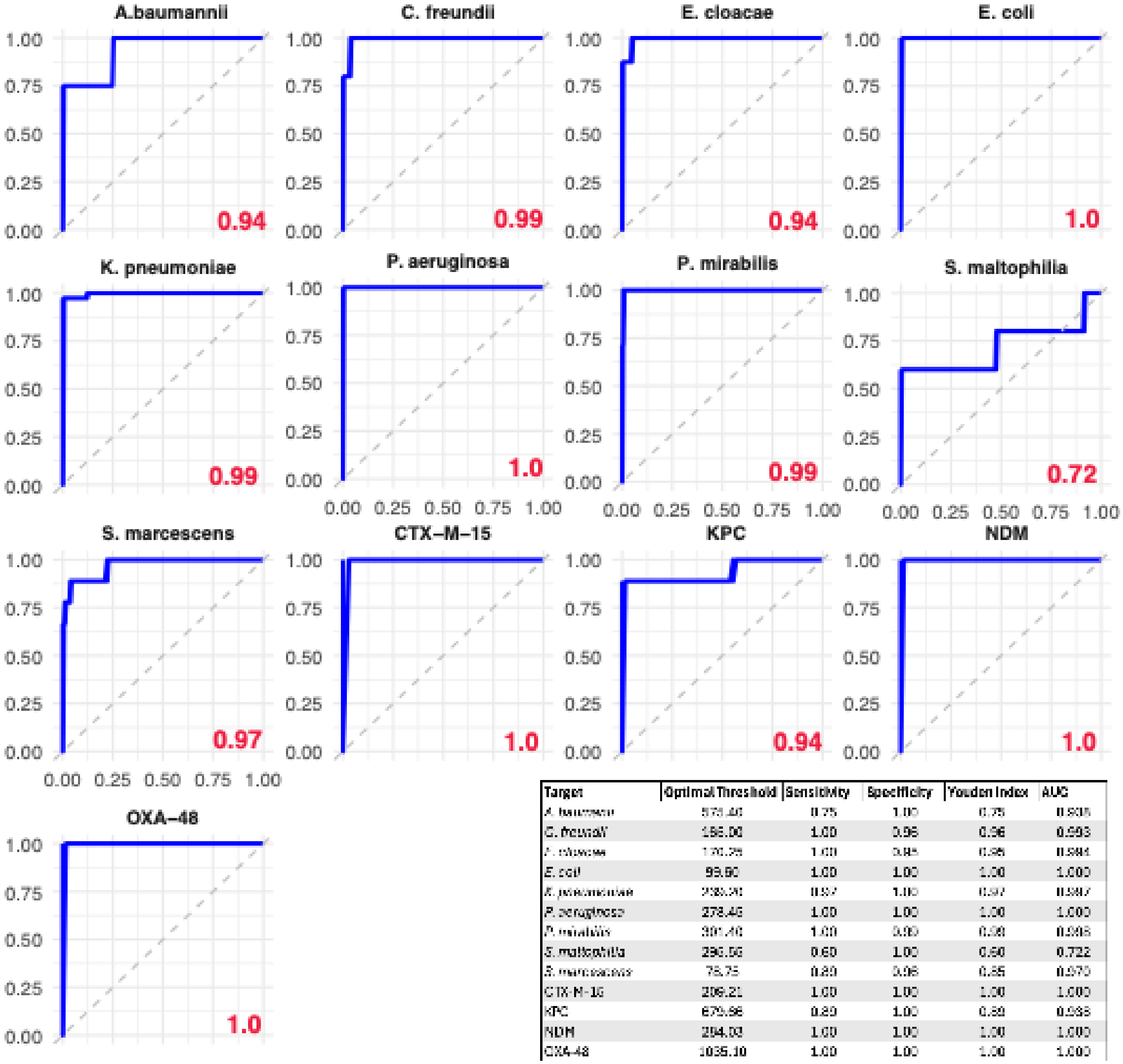

Target	Optimal Threshold	Sensitivity	Specificity	Youden Index	AUC
A. baumannii	573.40	0.75	1.00	0.75	0.938
C. freundii	186.00	1.00	0.96	0.96	0.993
E. cloacae	170.75	1.00	0.85	0.85	0.944
E. coli	99.90	1.00	1.00	1.00	1.000
K. pneumoniae	228.20	0.97	1.00	0.97	0.987
P. aeruginosa	278.46	1.00	1.00	1.00	1.000
P. mirabilis	301.40	1.00	0.98	0.98	0.993
S. maltophilia	296.56	0.60	1.00	0.60	0.722
S. marcescens	78.75	0.89	0.96	0.85	0.970
CTX-M-15	209.21	1.00	1.00	1.00	1.000
KPC	679.86	0.89	1.00	0.89	0.938
NDM	294.03	1.00	1.00	1.00	1.000
OXA-48	1035.10	1.00	1.00	1.00	1.000

Figure S9. Receiver operating characteristic (ROC) curves for each species-specific and AMR gene target. Curves were generated by comparing RFU values to known true-positive and true-negative classifications after excluding suspected contaminants (defined as samples with repeat RFU values >3-fold lower than the original) and *E. coli* false negatives lacking the *chuA* gene as confirmed by PCR. Each curve reflects the performance of a primer-guide target, with the area under the curve (AUC) in red text. Most targets demonstrated high discriminative ability, with AUC values ≥ 0.97 for the majority of species and AMR genes. The inset table displays the optimal threshold values along with their corresponding performance metrics across the cohort.

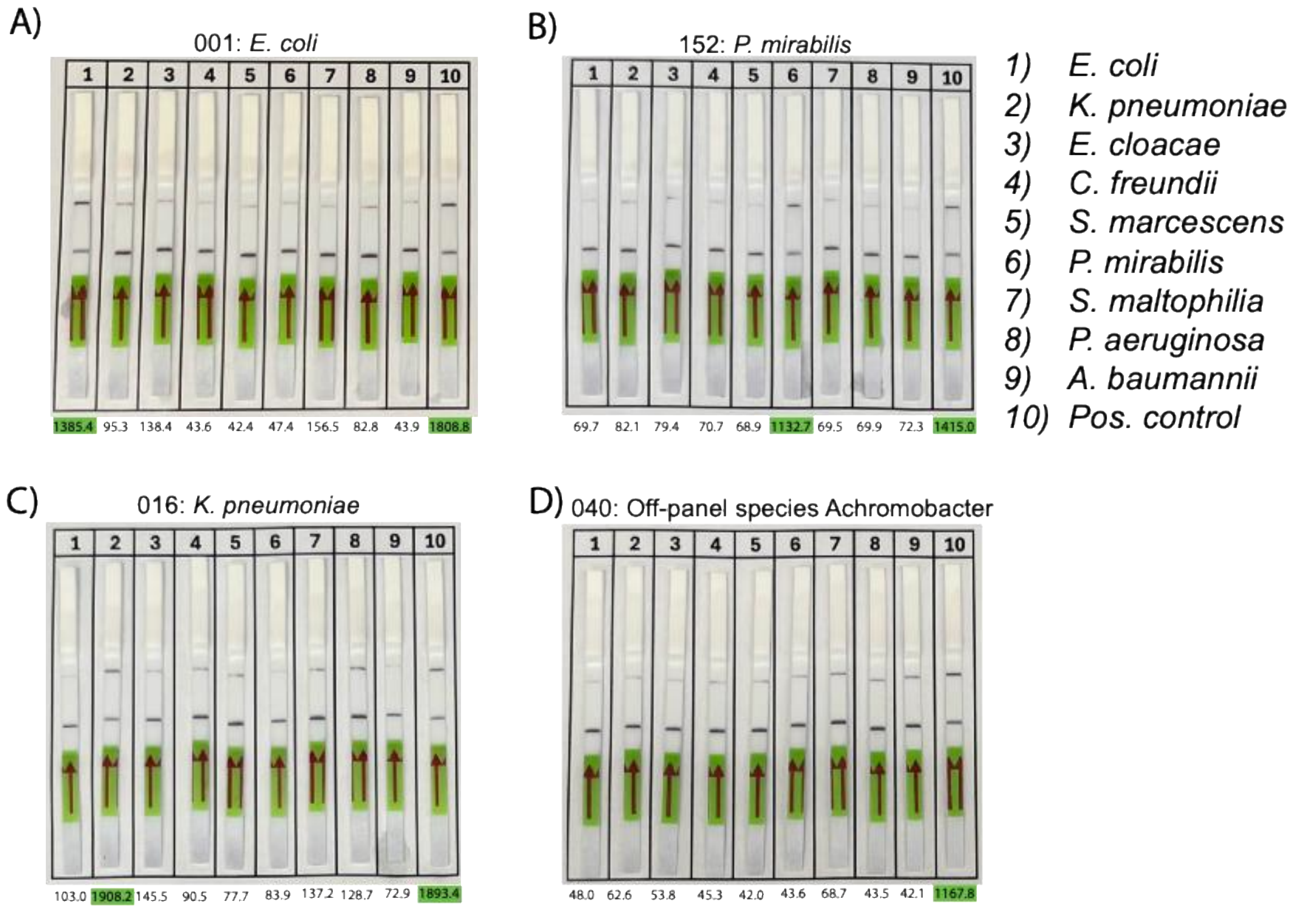

Figure S10. Lateral flow strips for several representative isolates, with the true species listed in the header. As in the fluorescent readout, each sample was run across all species targets in the order listed at right. RFU values from each BADLOCK reaction using the fluorescent readout are shown below, with true positive values highlighted in green.

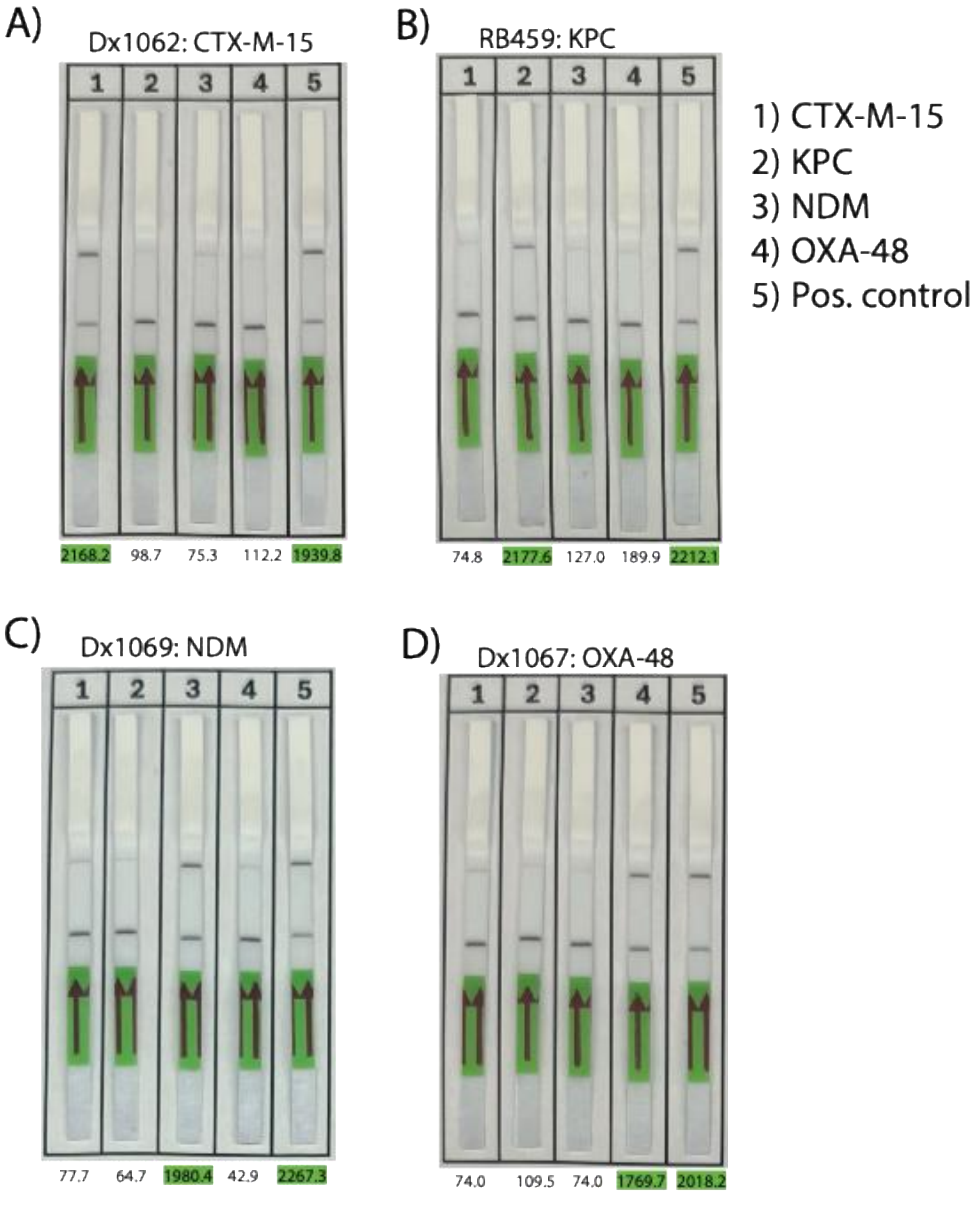

Figure S11. Lateral flow strips for AMR genes across different representative isolates, with the same format as above. RFU values from each BADLOCK reaction are shown below, with true positive values highlighted in green.

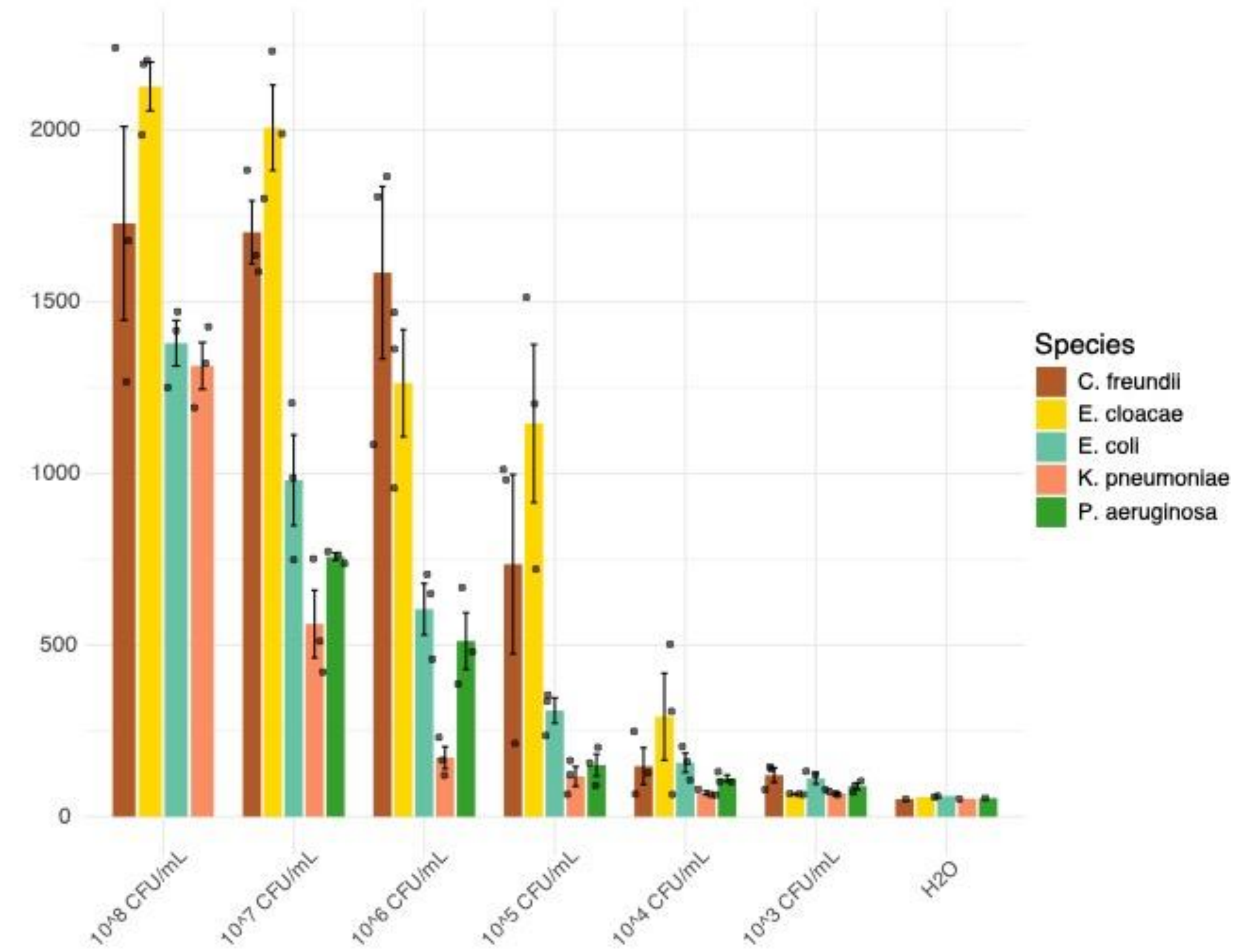

Figure S12. Limit of detection (LoD) for selected bacterial species included in the urine-based BADLOCK testing. Bars represent the lowest concentration (CFU/mL) at which each species was consistently detected using the BADLOCK assay. LoD values were determined using dilution series in pooled human urine, with detection defined as consistent assay positivity across replicates.

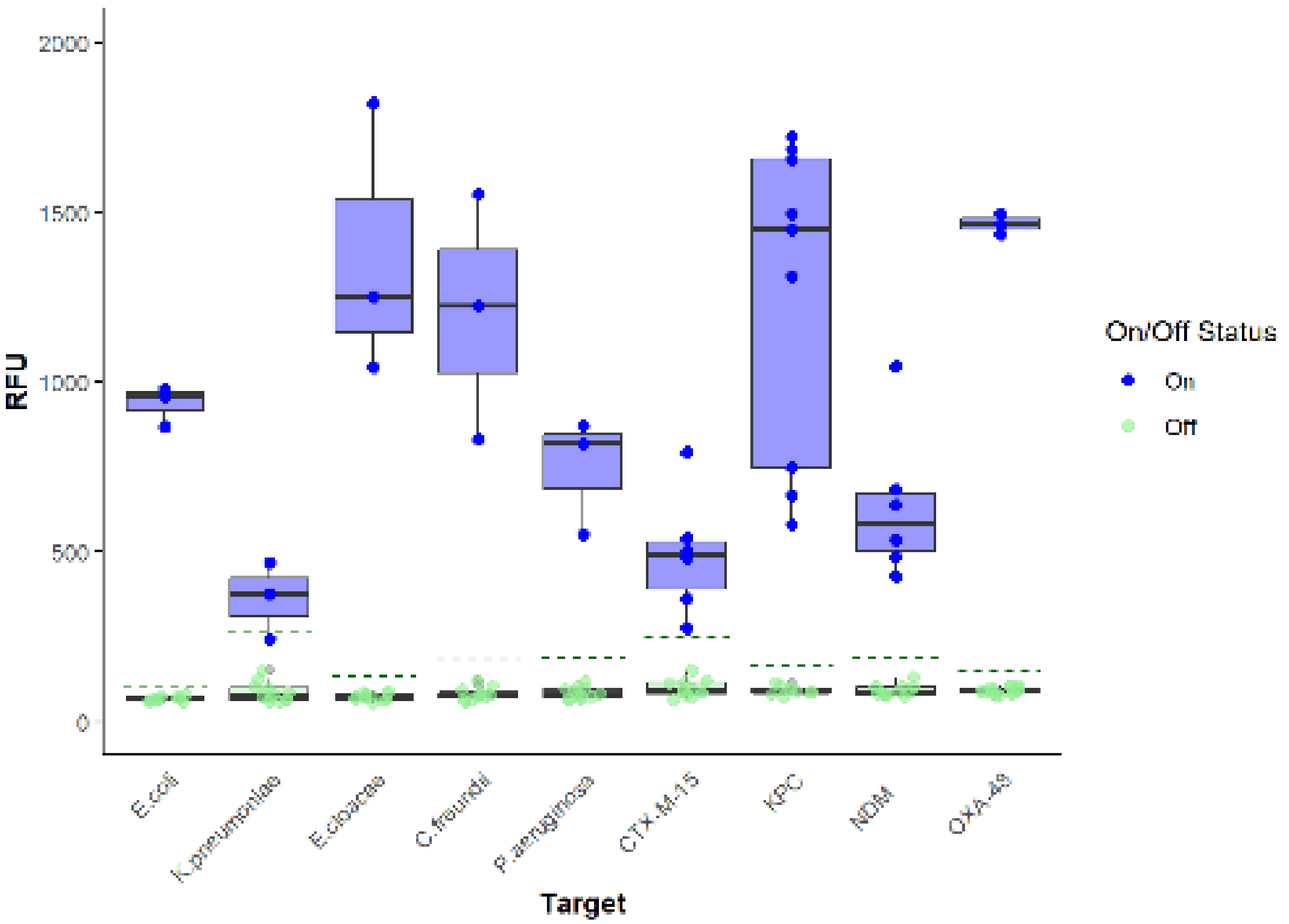

Figure S13. Threshold determination for species and antimicrobial resistance gene targets in the BADLOCK assay on commercial pooled human urine samples spiked with the indicated pathogen at 10^5 cfu/mL. Fluorescence values from technical replicates are shown. The horizontal dashed line indicates the threshold for calling a positive result, defined as the mean plus six times the standard deviation of fluorescence values from 12 off-target samples for species reactions and between 9 and 15 off-target samples for AMR gene reactions (variation due to some strains containing multiple target genes).

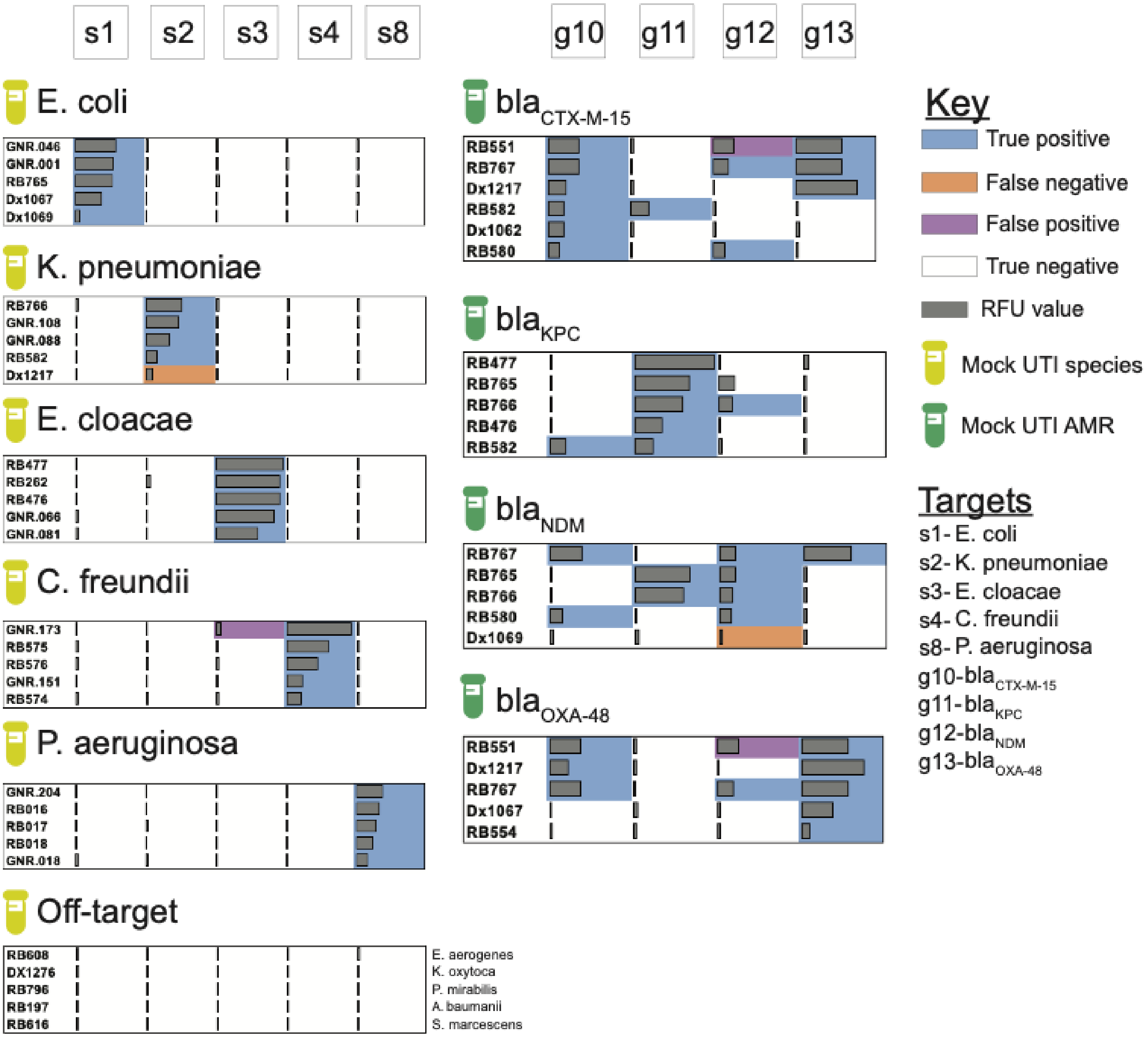

Figure S14. Consolidated visualization of BADLOCK assay runs for mock urinary tract infection (UTI) samples, including both species detection and AMR gene testing. Each row represents an individual sample, and each column corresponds to a BADLOCK target with the assay layout shown in the boxes above and listed in the accompanying key. Samples are grouped according to the species or AMR gene known to be present. Each cell in the grid reflects a single reaction for a given sample, color-coded by call, with overlaid grey bars representing the corresponding RFU values. Symbols adjacent to each target indicate the cohort or target class (species or resistance gene), harmonized with the legend in main-text Figure 3. Samples containing multiple targets are shown in each group for which a corresponding target was present. The off-target species are listed next to the sample row.

Species	Target Gene	RPA Forward	RPA Reverse	Guide
E. coli	*chuA*	gaaattaatacgactcactatagggATATGGCGGTGAGTATTATCGTCAGGAACAACATC	GAGATGACCATTTGTCGGCATCAACATCTTTGTAG	CGGCGCGACGACGGGCTTCCCGCAAGCAgttttagtccccttcgtttttggggtagtctaaatcccctatagtgagtcgtattaatttc
K. pneumoniae	*topA*	gaaattaatacgactcactatagggGGTGACGCCCTGCCGCTGCAGGTAACCCATAA	ATCTTCACGCTCCAGTACGCTGTAGCTGGCTTT	GTGATGAGACCATGGCGGCGGTATCGCTgttttagtccccttcgtttttggggtagtctaaatcccctatagtgagtcgtattaatttc
P. aeruginosa	*lasB*	gaaattaatacgactcactatagggATGTTCTATCCGCTGGTGTCGCTGGA	ACCGCTGCCCTTCTTGATGTCGTAG	TTCTATATGCGCGGCAAGAACGACTTCCgttttagtccccttcgtttttggggtagtctaaatcccctatagtgagtcgtattaatttc
E. cloacae	*contig23*	gaaattaatacgactcactatagggAATGGGCGTATGATTATACCCAGGTAA	AGCAGTTTGGCAAAGTGCGCTTGTGCAG	AGTCAACTATTAGGCCAAAGCTATGATCgttttagtccccttcgtttttggggtagtctaaatcccctatagtgagtcgtattaatttc
C. freundii	*topA*	gaaattaatacgactcactatagggGTTGAAAACCGTCGCTTTTACGCTGAAAAA	TCCATTCGGCTTCTTTATTCGCCACCTGGTC	ATGGGTGAAATCGTCACCGATCGTCTGGgttttagtccccttcgtttttggggtagtctaaatcccctatagtgagtcgtattaatttc
S. maltophilia	*400kb_contig*	gaaattaatacgactcactatagggGACCATCCTGTACGCGCTGGGCTGGACGCAGC	CACCACGATTTCATCAGGCTGACGTGGAACTTC	TGTCCGACCTGCTGCCCGGCTACCTGACgttttagtccccttcgtttttggggtagtctaaatcccctatagtgagtcgtattaatttc
P. mirabilis	*topA*	gaaattaatacgactcactatagggCGGTACGCCTACTCGTCGAGCGTGAACGTG	CGTCACTTCCATACGCAAGGGTTGTTCATC	ATTGGCAATTACACGCTTCACTGCTGACgttttagtccccttcgtttttggggtagtctaaatcccctatagtgagtcgtattaatttc
A. baumannii	*topA*	gaaattaatacgactcactatagggGGCACTTACGCGAAGTGATTGGTGGTGAC	CACAGTAATGGCGAAACCATGAAGCCTAC	CAGCCAACACGTCTCGACTTAAACCGTGgttttagtccccttcgtttttggggtagtctaaatcccctatagtgagtcgtattaatttc
S. marcescens	*luxS*	gaaattaatacgactcactatagggGAAGAGCAGCGCGTTGCCGATGCCTGGAAAG	CTTTCGGCAGCGCCAGTTCGTCGTTGTGGT	TGTGGTACCTACCACATGCACTCGCTGGgttttagtccccttcgtttttggggtagtctaaatcccctatagtgagtcgtattaatttc
NA	KPC	gaaattaatacgactcactatagggCGTCTAGTTCTGCTGTCTTGTCTCTCATGG	CAAAGTCCTGTTCGAGTTTAGCGAATGGTT	GCTGGCTGGCTTTTCTGCCACCGCGCTGgttttagtccccttcgtttttggggtagtctaaatcccctatagtgagtcgtattaatttc
NA	NDM	gaaattaatacgactcactatagggAATGTCTGGCAGCACACTTCCTATCTCGAC	TGATCTCCTGCTTGATCCAGTTGAGGATCT	CAACGGTTTGATCGTCAGGGATGGCGGCgttttagtccccttcgtttttggggtagtctaaatcccctatagtgagtcgtattaatttc
NA	CTX-M-15	gaaattaatacgactcactatagggATAAAACCGGCAGCGGTGGCTATGG	GCTAATACATCGCGACGGCTTTCTGCCTTA	ATCGTGCGCCGCTGATTCTGGTCACTTAgttttagtccccttcgtttttggggtagtctaaatcccctatagtgagtcgtattaatttc
NA	OXA-48	gaaattaatacgactcactatagggTTAAAATTCCCAATAGCTTGATCGCCCTCG	ATAAACAGGCACAACTGAATATTTCATCGC	CCAAGTCTTTAAGTGGGATGGACAGACGgttttagtccccttcgtttttggggtagtctaaatcccctatagtgagtcgtattaatttc
Synthetic target	NA	gaaattaatacgactcactatagggaggg	TAGTTATTGCTCAGCGGTGG	GGCCGTCCGTTAATTTCCCTTGCATACAgttttagtccccttcgtttttggggtagtctaaatcccctatagtgagtcgtattaatttc

Table S1. Primer and guide sequences for each target gene included in our panel. Lowercase letters indicate conserved sequences (T7 primer sequence for the forward RPA primer, and non-targeting region of the guide sequence, respectively), whereas uppercase are the target-specific component.

Species	True (+)	False (+)	True (-)	False (-)	Sensitivity	Specificity
E. coli	66	1	115	12	84.6	99.1
K. pneumoniae	36	0	155	3	92.3	100
E. cloacae	7	2	184	1	87.5	98.9
C. freundii	4	2	187	1	80	98.9
S. marcescens	7	4	181	2	77.8	97.8
P. mirabilis	7	4	183	0	100	97.9
S. maltophilia	3	1	188	2	60	99.5
P. aeruginosa	8	1	185	0	100	99.5
A. baumannii	3	4	186	1	75	97.9

Table S2. Performance characteristics for each species target.

Sample ID	Published ID	Source of Strain	Cohorts	Species	AMR genes
DX1003	MGH145	PMID: 28096418	AMR	*C. freundii*	KPC-2
DX1034	DH10Beta + pBAD_KPC3	Bhattacharyya Lab Strain	AMR	*E. coli*	KPC-3
DX1062	RB001+CTX-M-15	Bhattacharyya Lab Strain	AMR, Urine species	*E. coli*	CTX-M-15
DX1067	RB001+OXA-48	Bhattacharyya Lab Strain	AMR, Urine species, Urine AMR	*E. coli*	OXA-48
DX1068	RB059+OXA-48	Bhattacharyya Lab Strain	AMR	*E. coli*	OXA-48
DX1069	RB001+NDM-1	Bhattacharyya Lab Strain	AMR, Urine species, Urine AMR	*E. coli*	NDM-1
DX1217	AR0066 (CDC)	CDC AR Isolate Bank	AMR, Urine species, Urine AMR	*K. pneumoniae*	CTX-M-15, OXA-232
DX1218	AR0068 (CDC)	CDC AR Isolate Bank	AMR	*K. pneumoniae*	CTX-M-15, NDM-1, OXA-48
DX1219	AR0143 (CDC)	CDC AR Isolate Bank	AMR	*K. pneumoniae*	CTX-M-15, NDM-1
DX1220	AR0153 (CDC)	CDC AR Isolate Bank	AMR	*K. pneumoniae*	CTX-M-15, NDM-1, OXA-48
DX1276	MGH175	PMID: 28096418	Urine species	*K. oxytoca*	na
RB016	2732233	Bhattacharyya Lab Strain	Urine species	*P.aeruginosa*	na
RB017	B895023	Bhattacharyya Lab Strain	Urine species	*P.aeruginosa*	na
RB018	2762085	Bhattacharyya Lab Strain	Urine species	*P.aeruginosa*	na
RB197	Acinetobacter #1	Bhattacharyya Lab Strain	Urine species	*A. baumanii*	na
RB262	SBJ-32413; 1349	Bhattacharyya Lab Strain	Urine species	*E. cloacae*	na
RB459	BIDMC68	PMID: 28096418	AMR	*K. pneumoniae*	KPC-2
RB461	MGH67	PMID: 28096418	AMR	*K. pneumoniae*	KPC-2
RB462	MGH71	PMID: 28096418	AMR	*K. pneumoniae*	KPC-2
RB476	BWH39	PMID: 28096418	AMR, Urine species, Urine AMR	*E. cloacae*	KPC-2
RB477	MGH5	PMID: 28096418	AMR, Urine species, Urine AMR	*E. cloacae*	KPC-2
RB551	BWH2	PMID: 28096418	AMR, Urine AMR	*K. pneumoniae*	CTX-M-15, OXA-48
RB554	BAA2524	PMID: 28096418	AMR, Urine AMR	*K. pneumoniae*	OXA-48
RB574	MGH281c	PMID: 28096418	Urine species	*C. freundii*	na
RB575	MGH281	PMID: 28096418	Urine species	*C. freundii*	na
RB576	MGH283	PMID: 28096418	Urine species	*C. freundii*	na
RB579	IDR1600031102-01-00	Wadsworth Lab	AMR	*K. pneumoniae*	CTX-M-15
RB580	IDR1600014966-01-00	Wadsworth Lab	AMR, Urine AMR	*Citrobacter spp*	CTX-M-15, NDM-1
RB582	IDR1600037319-01-00	Wadsworth Lab	AMR, Urine species, Urine AMR	*K. pneumoniae*	CTX-M-15, KPC-2
RB608	BAC0800002538	Wadsworth Lab	Urine species	*E. aerogenes*	na
RB616	IDR1300010999	Wadsworth Lab	Urine species	*S. marcescens*	na
RB765	F3461517	PMID: 28096418	AMR, Urine species, Urine AMR	*E. coli*	KPC-2, NDM-1
RB766	F3321027	PMID: 28096418	AMR, Urine species, Urine AMR	*K. pneumoniae*	KPC-2, NDM-1
RB767	X1621267	PMID: 28096418	AMR, Urine AMR	*E. coli*	CTX-M-15, NDM-1, OXA-48
RB796	MGH013	PMID: 28096418	Urine species	*P. mirabilis*	na

Table S3. Banked clinical isolates with known genomic content. The table includes the testing cohort(s) in which each sample was used, the species name, and the relevant antimicrobial resistance gene(s) of interest.
